# P-410. Gastrointestinal multi-drug resistant bacterial tango in Singapore General Hospital

**DOI:** 10.1093/ofid/ofae631.611

**Published:** 2025-01-29

**Authors:** Indumathi Venkatachalam, May Kyawt Aung, Mabel Z Q Foo, Yong Yang, Deborah C M Lai, Jean Xiang Ying Sim, Moi Lin Ling

**Affiliations:** Singapore General Hospital, Singapore, Singapore; Singapore General Hospital, Singapore, Singapore; Singapore General Hospital, Singapore, Singapore; Singapore General Hospital, Singapore, Singapore; Singapore General Hospital, Singapore, Singapore; Singapore General Hospital, Singapore, Singapore; Singapore General Hospital, Singapore, Singapore

## Abstract

**Background:**

The gastrointestinal tract (GIT) is an ideal reservoir of antibiotic resistance genes where, under antibiotic pressure, multidrug resistant organisms (MDROs) express and disseminate resistance genes via horizontal gene transfer. Control of GIT-MDROs such as vancomycin resistant *Enterococci* (VRE) and carbapenemase producing *Enterobacterales* (CPE) within healthcare-settings remains challenging.

VRE Epidemiology

**Figure:**
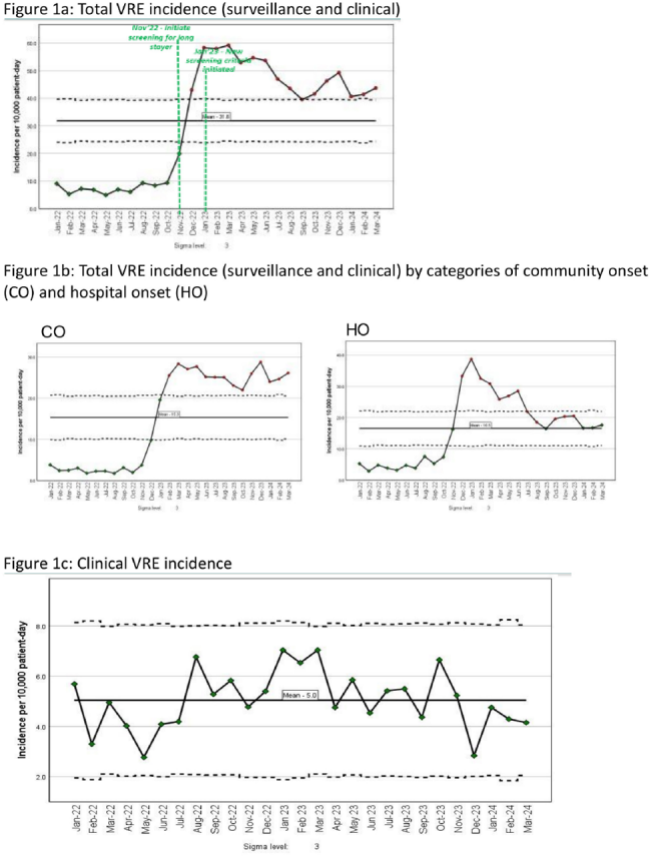

**Methods:**

In Singapore General Hospital (SGH), a 2000-bed tertiary healthcare institution, vertical infection prevention programs have been implemented for VRE and CPE control. Selected high risk patients undergo rectal swabs or stool sampling for VRE and CPE genes. Positive samples taken less than three days after admission are considered community onset (CO),others are hospital onset (HO). Data between January 2022 and March 2024 were reviewed.

CPE Epidemiology

**Figure:**
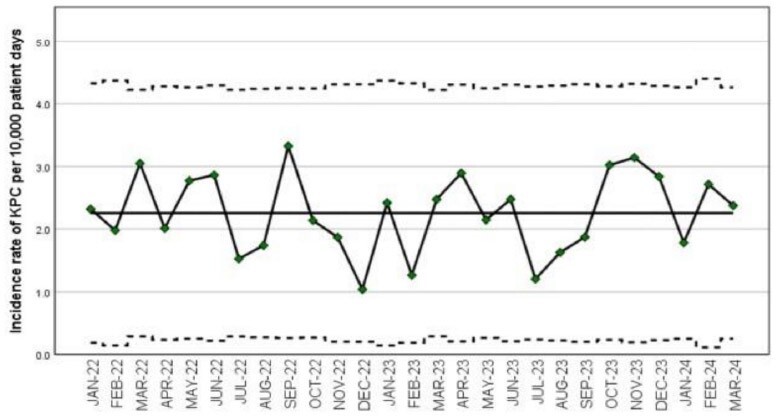

**Results:**

Burden of VRE total (surveillance and clinical) increased from a baseline of 40 cases per month (10 per 10,000 patient-days) to 200 per month (45 per 10,000 patient-days) after December 2022 when VRE surveillance was expanded (Figure 1a). CO-VRE total remained elevated but HO-VRE total reduced to a baseline of 20 per 10,000 patient-days after August 2023 (Figure 1b). Clinical VRE incidence was stable at 5 per 10,000 patient-days (Figure 1c). Median days to clinical VRE was 14 (1-250 days), 425 of 671 (63%) VRE clinical isolates were from urine. VRE acquisition was 30-35%.

Burden of CPE total (surveillance and clinical) has been stable at 120 cases per month with 57% being *Klebsiella pneumoniae* carbapenemase (KPC) type CPE (Figure 2a). Incident KPC total has been 90 per month (18 per 10,000 patient-days) (Figure 2b). CO-KPC total incidence increased from 4 per 10,000 patient-days in 2022 to 8 per 10,000 patient-days in 2023/2024 whilst HO KPC total remained stable (Figure 2c). Clinical KPC incidence was stable at 2 per 10,000 patient-days (Figure 2d). Median days to positive clinical KPC was 11 (1-204 days). 162 of 300 (54%) KPC clinical isolates were from urine. KPC acquisition was 30-40%.

**Conclusion:**

Trend of the total burden of VRE and KPC, corresponds to active surveillance strategy butI clinical incidence has been stable over the 27-months. VRE and KPC acquisition remains high at 30-40%.

**Disclosures:**

**Moi Lin Ling, FRCPA**, Solventum: Honoraria|Solventum: Educational grant for APSIC projects

